# Role of birth companion in COVID-19: indispensable for her and an auxiliary hand for us

**DOI:** 10.11604/pamj.2020.37.62.23565

**Published:** 2020-09-15

**Authors:** Priyanka Kathuria, Ambica Khetarpal, Pratibha Singh, Shruti Khatana, Garima Yadav, Navdeep Kaur Ghuman

**Affiliations:** 1 All India Institute of Medical Sciences, Jodhpur, India,; 2 Khetarpal Diagnostics, Delhi, India,; 3 Adesh Medical College, Haryana, India

**Keywords:** COVID-19, pregnancy, birth companion

## Abstract

The pandemic of COVID-19 has proved to be a global catastrophe. Pregnant females could be more vulnerable to the infection owing to the immune modulation. According to the World Health Organization (WHO), pregnant females including those with COVID-19 suspicion or confirmed status have right to 'safe and positive childbirth experience' which includes a companion. The birth companion, is present at all times with the patient, from the initiation of labor till breastfeeding. The COVID-19 crisis has taken its toll on the healthcare system. The number of infected antenatal females are expected to increase. If a birth companion is trained in basic intrapartum and postpartum observation and care, he/she can be utilised to minimize unnecessary patient-clinician interface and optimize manpower in this critical time.

## Commentary

**Introduction:** the pandemic of COVID-19 has proved to be a global catastrophe. The course of this disease is uncertain since the causative agent is novel (SARS-CoV-2). Amongst others, pregnant females could be more vulnerable to the infection owing to the immune modulation. WHO has urged that all antenatal females, with suspected/confirmed COVID-19 status, have the 'right to high quality care' which amongst other privileges includes having a companion of choice during delivery [1]. A labor companion ameliorates outcome for the female and the baby (Cochrane systematic review) [2]. A labor companion supports the female physically, psychologically and emotionally.

**Invisible auxiliary hand for us:** with the burgeoning number of COVID infected patients, the number of available healthcare professionals isn´t large enough to lavish significant attention to each stricken patient. As they not only look after the patient but are also involved in finding cases, tracing contacts and providing public health information to masses. Normal labor is a time consuming process and demands regular monitoring, especially in a COVID-19 suspected/confirmed patient. It may be difficult to appoint a healthcare professional per laboring patient. Since the birth companion will be staying with the patient, from initiation of labor till breastfeeding, she/he is the ideal person who can be trained to take the load off the healthcare worker´s shoulders by monitoring and taking care of laboring patients, both intrapartum and postpartum.

**Significance of a birth companion in this COVID-19 crisis:** in the light of knowledge available till date, regarding SARS-CoV-2, the importance of birth companion cannot be overemphasized: 1) Most of the SARS-CoV-2 positive females have delivered by Caesarean section till date [3]. For early recovery ERAS guidelines are being followed. This recommends a regular diet within 2 hours, early mobilization and urinary catheter removal immediately after caesarean delivery [4]. This mayn´t be possible for the female without assistance. 2) Shortening of the second stage of labour with elective instrumental birth, in selected cases, is being recommended for COVID-19 patients [5]. In such cases larger episiotomy is the cause of discomfort and warrants assistance. 3) Transmission of COVID-19 in breast milk has not been documented [3,6]. The huge benefit of breast milk cannot be underestimated especially in low resource countries. Breast milk may contain protective factors after maternal COVID-19. Hence this may add to the other well established benefits of breast milk [6]. 4) The decision for separating the baby or rooming in should be taken after discussion with the patient. The benefit of temporary separation for prevention of aerosol transmission must be discussed with her in labour itself. The benefits of separation may be greater in mothers with more serious illness [6]. An assistant is essential to take care of the baby, the companion also helps in sterilising the equipment and milk expression by the mother. 5) The mental status is quite vulnerable for a mother who is suffering or is recovering from an acute illness. Nonetheless, isolation from the infant (if done) adds to the mother´s suffering, putting her at risk of developing anxiety or postpartum depression. A birth companion would support her psychologically and emotionally by listening to her, keeping her calm, empathising, helping her connect to information on her exact condition and recovery.

**Prerequisites, precautions and management of a birth companion:** the following aspects were specifically taken care of, in context of prerequisites, precautions and management of a birth companion for a female with suspected/confirmed COVID-19 infection: 1) The female must identify a trustworthy birth companion beforehand and a potential alternative, should the need arise [5]. 2) At the time of admission or whenever she goes into labour, the clinical situation of the patient must be explained to the birth companion and his/her willingness to opt in must be ascertained. 3) A written informed consent must be taken from the birth companion which must include the statement that the decision is voluntary, explains the probable chances of COVID-19 infection and its consequences and acceptance to follow the recommended precautions to avoid infection. 4) At the same time, the birth companion must be asymptomatic.

**Figure 1:**
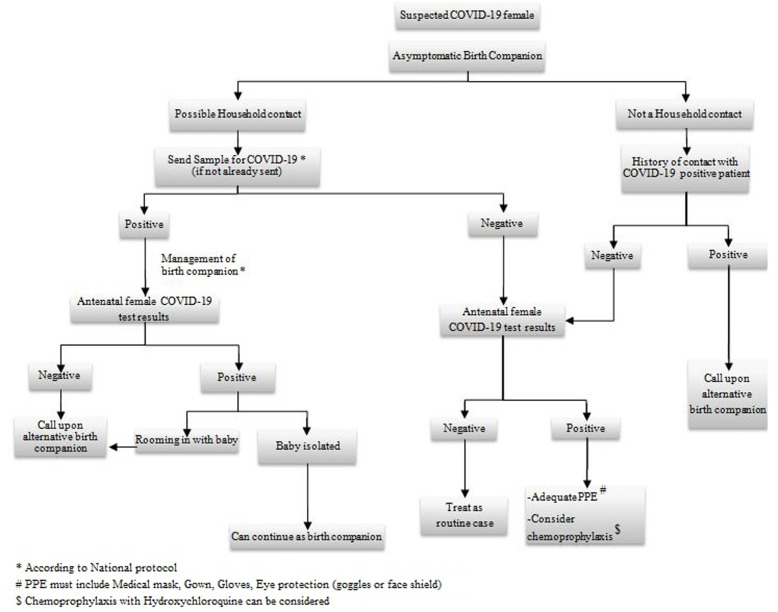
the protocol for selection, precautions and management of birth companion in a COVID-19 suspected antenatal female

**Figure 2:**
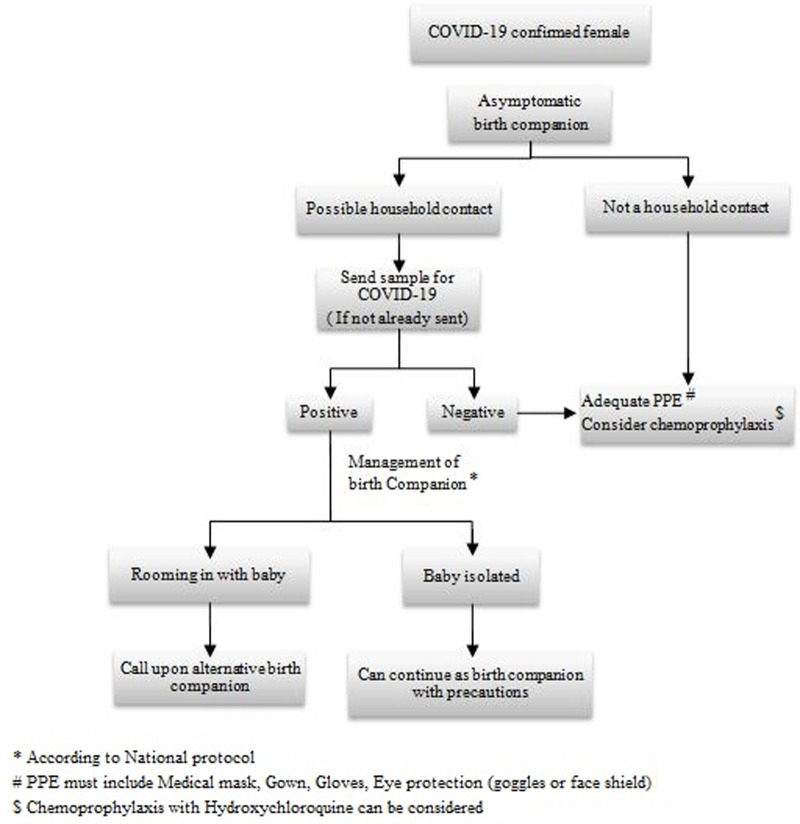
the protocol for selection, precautions and management of birth companion in a COVID-19 confirmed antenatal female

**Protocol developed:** Figure 1 elaborates the protocol for selection, precautions and management of birth companion in a COVID-19 suspected antenatal female [7] while Figure 2 explains the protocol for selection, precautions and management of birth companion in a COVID-19 confirmed antenatal female [8]. During the drafting of the protocol for selection, precautions and management of birth companion, it came to light that the household to which the birth companion belonged to, formed the most significant factor. COVID-19 testing will be taken for the birth companion identified by the female, if he/she is a possible household contact (definition of contact as per WHO) [9]. The birth companion, if found positive has to be managed according to the National protocol. If an antenatal female who was diagnosed with COVID-19 earlier in pregnancy and has got a negative report before going into labor, then she is considered as a routine antenatal patient, and any asymptomatic birth Attendant can be selected [10]. Post care isolation must be considered in all birth companions. The duration may vary depending upon their symptoms, signs, clinical findings and the National protocol.

**Training of birth companion:** for training of the birth companion, the following must be considered: i) Females who already have children make a good choice. ii) The birth companion will be staying with the female during labour, therefore he/she could be helpful in observing the parameters during labour. Since these are not extremely technical, they can be easily taught beforehand. This would significantly decrease the burden on healthcare workers. iii) Written instructions and videos (including mock drill videos) containing details on Do´s and Don´ts to prevent COVID-19 infection, in addition to the routine information for a birth companion should be provided beforehand. This should include the following details of: a) General precautions including distancing and providing only the essential care. b) Hand hygiene. c) Techniques to record temperature (thermometer), respiratory rate (count), oxygen saturation and pulse rate (pulse oximeter) during intrapartum and postpartum period. d) Significant values of the above parameters for notification to the healthcare worker. e) Use of electronic media for communication with the health worker. f) Linen changing for mother and baby. g) Precautions while collecting and sending laundry for both, the mother and the baby. h) Waste disposal. i) Distancing the baby while not feeding. j) Prevention of aerosol exposure for baby while feeding. k) Helping milk expression. i) Methods for sterilisation of bottles, pumps etc. m) Safeguarding her mental health.

## Conclusion

The total number of COVID-19 cases, surfacing each day is still increasing. In such an upsetting scenario, more number of antenatal females are expected to get infected. Care of an antenatal female in labor demands not just an obstetrician but the entire team. This means that additional number of health providers would be exposed to the virus. In this race against the clock, to halt this adversity, utilizing the help of a birth companion would be extremely resourceful. As we traverse these unchartered territories in times of COVID-19 crisis, the optimum utilization of a birth companion can immensely reduce the load on healthcare workers, minimize unnecessary patient-clinician interface and can be a boon for the hospitals to optimize manpower in times of crisis.
